# Virtual conference design: features and obstacles

**DOI:** 10.1007/s11042-022-12402-4

**Published:** 2022-03-03

**Authors:** William Hurst, Adam Withington, Hoshang Kolivand

**Affiliations:** 1 Information Technology Group, Wageningen University and Research, Leeuwenborch, Hollandseweg 1, 6706 KN Wageningen, Netherlands; 2 Engineering and Technology, Liverpool John Moores University, Byrom Street, Liverpool, L3 3AF UK; 3 Department of Computer Science, Liverpool John Moores University, Byrom Street, Liverpool, L3 3AF UK

**Keywords:** Virtual conferencing, User experience, Covid-19, User journey mapping

## Abstract

The Covid-19 pandemic has forced a change in the way people work, and the location that they work from. The impact has caused significant disruption to education, the work environment and how social interactions take place. Online user habits have also changed due to lockdown restrictions and virtual conferencing software has become a vital cog in team communication. In result, a spate in software solutions have emerged in order to support the challenges of remote learning and working. The conferencing software landscape is now a core communication solution for company-wide interaction, team discussions, screen sharing and face-to-face contact. Yet the number of existing platforms is diverse. In this article, a systematic literature review investigation on virtual conferencing is presented. As output from the analysis, 67 key features and 74 obstacles users experience when interacting with virtual conferencing technologies are identified from 60 related open-source journal articles from 5 digital library repositories.

## Introduction

**Fig. 1:**
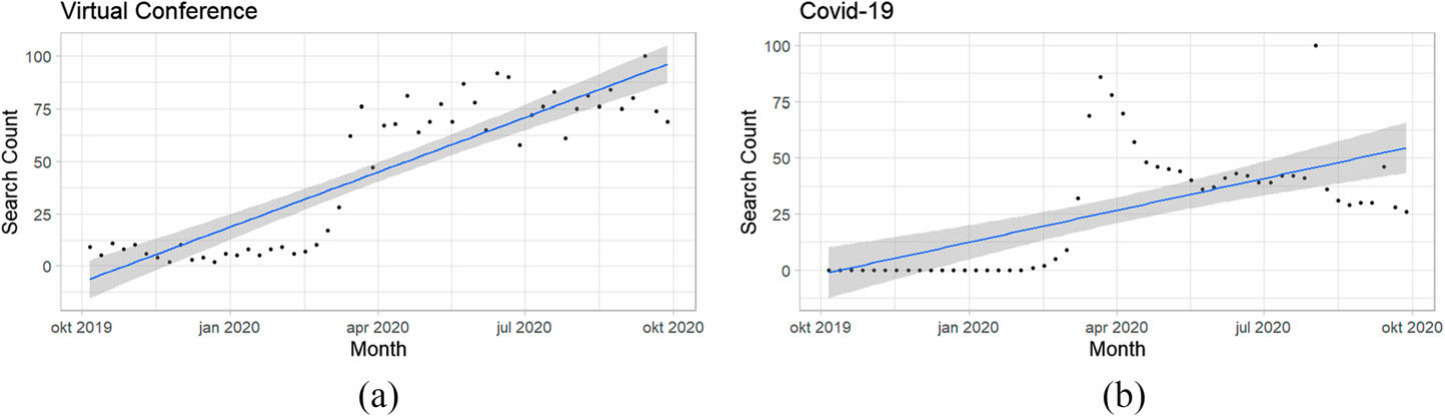
Google Trends from Oct 19 to Oct 20 with **a** depicting virtual conference searches and **b** Covid-19 searches

In April 2020, 46.6% of employees were estimated to be working from home during the first wave of the UK Covid-19 pandemic [9]. An increase in digital meetings became a feature of the 2020 work environment with a growing role for virtual conferences [52] and a new range tools emerged to support the transition from the workplace to the home setting [10]. Yet, this move towards a conference virtualisation was already in motion prior to the pandemic. For example, as Forbes et al. outline, in the US, workforces have become more distributed in recent years with 55% of companies already enabled for remote working prior to the pandemic (with 30% previously working remotely) [7, 27, 59]. Examples have emerged of businesses that have found working from home to be a way of reducing expenditure (e.g. reduced rent and maintenance costs) [61] and improve staff productivity [14], in some cases by up to 77% [59]. The effect of Covid-19, however, produced an uncalculated sharp increase in remote working within a shorter space of time than expected with the pandemic acting as a catalyst for an already increasing trend. This sharp increase is evident in the Google search patterns from October 2019 to October 2020 displayed in Fig. 1, demonstrating this sudden inflated interest in virtual conferencing solutions. Search terms *Virtual Conference* and *Covid-19* are depicted, where a value of 100 is the peak popularity. A sustained increase in searches for Virtual Conference remains, continuing in an upward trend beyond the first UK lockdown, whereas a *Covid-19* searches are later on the decline.

Aside from affecting the collaborative team working environment, the move to virtual conference settings have had a wider impact across multiple sectors, with corporations unable to showcase products and innovations within expos. Similarly, with universities unable to conduct standard student open days, they have adjusted by means of virtual solutions. For example, TU Delft recreated their campus using Minecraft^1^ to produce a virtualisation of their university grounds to enable students to remotely explore the environment and gain a more in-depth impression of the student setting [11, 16].^2^ Other universities have also employed this approach, with a list of virtual tours and videos collated and presented by the Universities and Colleges Admissions Service (UCAS) [68]. As of November 2020, a total of 122 UK-based universities adopted a virtual campus methodology (e.g. interactive maps with 360-degree video capture to create a virtual tour environment, or high-quality video production tours). Yet, so far, there is no unified virtual conference solution employed for product, location or exposition showcasing.

Creating the ideal virtual environment in which participants can communicate effectively remains a prevalent challenge for designers. The notion of culture and having a physical meeting place is something virtual conferences will always struggle to replace. One potential solution to this is to produce ultra-realistic virtual conference environments in 3D, providing a more augmented experience using game engine technologies coupled with headsets and other hardware solutions. To-date, 3D-virtual applications have been proven to be an effective metric for showcasing digital heritage [12] for use with virtual tourism-based applications in particular. Digitising cultural heritage provides sustainability, a way to engage with the public for the sharing of knowledge and virtual tourism opportunities. It is possible that this technology (i.e., game engines) can cater for close-to-real-life environment and interactions. Yet, access to 3D models and digital content is often a limitation, affecting the quality of the production [12] and future digital conference platforms need to recognise the challenge surrounding 3D asset inclusion in the development of a scalable, and engaging environment. For instance, the quality and level of detail (e.g. polygon count) can reduce functionality, despite achieving impressive realism, with multiple users interacting within the environment. Game assets should adhere to low polygon counts, to reduce render times and increase framerate; particularly in real-time expositions. This approach would also require an extensive Infrastructure as a Service (IaaS) to cater for the real-time engagement with the environment and support the scalability for multiple users. This is confirmed by Zhang et al. and Soltanian et al., who emphasise that a suitable cloud infrastructure network is often an issue for multi-source multimedia conference systems [58, 74]. This has led to researchers adopting existing infrastructures, such as Second Life to implement immersive environments as potential virtual conference solutions. August et al., for example, establish a virtual engineering lab that caters for interactive learning through visualisation and problem solving within the virtual Second Life world [5]; thus bypassing the need for creating a new cloud infrastructure.

Despite the sudden growth in the use of virtual conference technologies, it is essential that emerging virtual conference platforms are developed with full consideration of the features and obstacles regarding their design to meet the varied needs of their users. Therefore, in this article, an investigation into the related open source articles that focus on virtual conference solutions is presented. The findings are intended to showcase the features and obstacles associated with existing virtual conference solutions in current literature, specifically from 60 open-source journal articles. The remainder of this article is subsequently organised as follows. Section 2 provides outlines the methodology adopted in for the SLR. Section 3 outlines the results achieved and answers the defined research questions presented in Section 2. The article is concluded in Section 4.

## Methodology

Beyond the need for academic communication and expositions, virtual conferences also serve as suitable training and skills development environments. As Lowe et al. discuss, virtual environments have the potential to be widely adopted for disaster readiness training and education [39]. In their investigation, they document the feasibility of 360 VR technology for adolescent disaster readiness. Yet, the challenge with VR technology, as discussed with by the authors, is that there is a learning curve associated with the use of VR technology headsets and hand controls. For a wider deployment of the technology, for example in a virtual conference setting, all participants would need access to the hardware; meaning there are technological barriers. A solution would be to encourage users to attend the virtual conference on their smart phones. Modern-day smart phones are capable of catering for VR technology when coupled with polarised glasses. For example, Tregillus et al. outline that smart phones adaptors enable VR applications to be available for mass audiences. However, there are limitations surrounding interacting with the environment as users are constrained to head movements and are unable to perform hand-based locomotion [64]. Meaning designers must factor in that individuals may not be able to move around and navigate virtual environments. As Mohatta et al. discuss, the future of user interfaces will be governed by hand gestures, and therefore alternative hand gesture techniques are required for integrations with mobile technologies [43].

As highlighted in the related work, there is a willingness to change the way in which conferences take place. There is also a level of existing technology which is capable of supporting this transition. However, a suitable design framework is required for the production of the next generation of virtual conference solutions. In order to contribute to its development, a systematic literature review is conducted on digital conference and digital user experience design works by means of a quality assessment review process. The need for digital inclusion and the growing use of immersive technologies (e.g. augmented and virtual reality) has evolved the variety and functionality of virtual conference solutions. Usability is an indispensable consideration for virtual conference software developers, particularly for cyber learning environments [2]. During a pandemic period that has seen an increasing interest in the development of communication solutions, there must be a corresponding growth in understanding the positive (features) and negative (obstacles) experiences end-users have when using a digital virtual conference product.

### Systematic literature review

The systematic literature review (SLR) method adopted for the investigations is an adaptation of model employed by Tummers et al., in [66]. The approach aims to assess available research relevant to the investigation by undertaking six steps within a defined protocol, as follows.

#### Research questions

Based on the related works investigation in 2.1, the investigation will consider all domains in which virtual conferences are used. Specifically, the following questions are identified for the SLR process: Q1: *What are the features and obstacles of current virtual conferencing platforms*; Q2: *What are the user experience considerations*? and Q3: *What are the digital considerations for 3D virtual conference applications*?

#### Search strategy

A systematic search is conducted, focusing on open-source articles available in the IEEE Xplore, MDPI, Elsevier, Springer, Wiley digital libraries (ACM was also considered as a digital library source but was eliminated as, at the time of writing this article, it was not possible to filter articles by open access). The following search queries are conducted. 1) “Virtual Conference (or Digital Conference, or Digital/Virtual Workshop”; 2) “Digital User Experience”; and 3) “3D Conferencing (or Immersive Conferencing)”. Due to technology developments within the virtual conferencing domain, only articles from 2016 onwards are considered in the search results. The following pseudocode outlines the search queries employed.

1 $$ {\displaystyle \begin{array}{c}\left(\right(\left(\right(\left(\left(`` All\  Metadata": Virtual\ Conference\right)\  OR`` All\  Metadata": Digital\ Conference\right)\  OR`` All\ \\ {} Metadata": Virtual\ Expo\left)\  OR" All\  Metadata": Digital\ Expo\right)\  OR" All\  Metadata": Virtual\ Workshop\Big)\\ {} OR" All\  Metadata": Digital\ Workshop\Big)\end{array}} $$

2 $$ \left(\left(\left(`` All\  Metadata": Digital\right)\  OR`` All\  Metadata": Virtual\right)\  AND`` All\  Metadata": User\ Experience\right) $$

3 $$ \left(\left(`` All\  Metadata":3D\  OR\ Immersive\right)\  AND`` All\  Metadata": Conferencing\right) $$

#### Study selection criteria

As outlined in Table 1, the selection criteria are applied by a combination of filtering the search then examining the meta data accompanying the publications, reading the title, abstract and conclusion. For Search Criteria 3 (SC3), some search results generated overview documents after the first two stages (e.g. conference or workshop proceedings overviews, or introductions to special issues in journals) rather than full journal articles. This required manual exclusion.

**Table 1:** Selection Criteria (SC)

Code	Criteria
SC1	Year (published 2016 onwards).
SC2	Journal Article
SC2.1	Full Text
SC2.2	Written in English
SC2.3	Open Access
SC3	Provides a valid study (e.g. not a foreword or introduction document etc.)
SC4	Related to digital virtual conferencing

**Fig. 2:**
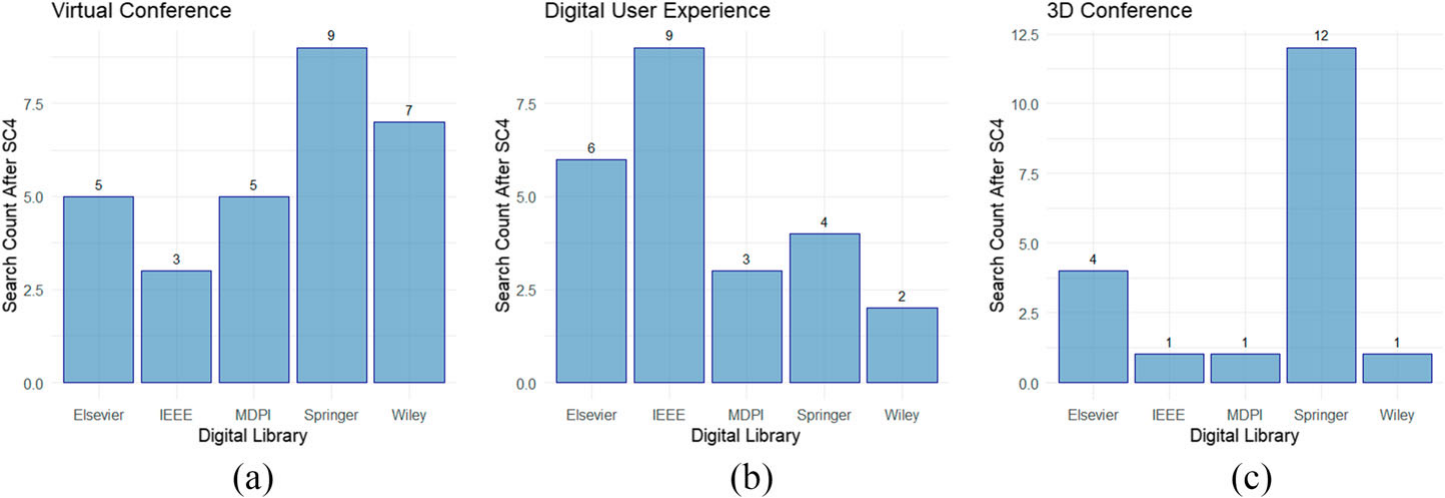
Articles Selected for Quality Assessment **a** Virtual Conference, **b** Digital User Experience and **c** 3D Conferencing

For Search Criteria 4 (SC4), research articles that provide a tool to enable virtual conferencing, even in a one-to-one capacity, are also included (e.g. virtual psychiatry [49], virtual learning [60] etc.). Figure 2 displays the articles selected for quality assessment per search query (i.e. 1–3 listed in Section 2.2.2 above) for each of the digital libraries.

#### Quality assessment

Following SC4, 72 studies are included for the quality assessment process, which is a manual procedure involving reading each of the publications and scoring by means of the quality criteria detailed in Table 2. Points are assigned to the article for providing a valid study (QA1), having clear documentation of methodology (QA2), clear documentation of findings (QA3), conclusion relates to study aims (QA4), overall quality for example clarity, within scope, valid and reliable results (QA5) and relevance to the investigation (QA6).

**Table 2:** Quality Assessment

Code	Criteria
QA1	Defined and valid study
QA2	Clear documentation of methodology
QA3	Clear documentation of findings
QA4	Does the conclusion relate to study aims.
QA5	Quality (e.g. journal ranking)
QA6	Discusses/Implements Virtual Conferencing Solutions

For the scoring of the criteria, the grading system employed by Tummers et al., in [66] is adopted. In this approach, points are awarded to each criteria on a scale of 1, 0.5 and 0; with 1 referring to the highest and 0 the lowest. A score of 0.5 is given if a criteria is somewhat met. As in [66], articles with a total score of <3 were excluded from the data extraction and synthesis stages. This meant that 12 studies are excluded prior to the data extraction phase. The selection criteria filtering and quality assessment count is displayed in Tables 3, 4 and 5.

**Table 3:** Virtual Conference Search

Digital Library	After Search	SC1	SC2	SC3	SC4	QA
IEEE	291,510	64,784	642	40	3	3
MDPI	239	209	209	209	5	3
Elsevier	1876	1577	76	76	5	4
Springer	1748	1122	64	52	9	6
Wiley	22,626	6272	323	283	7	7
Total	317,999	73,964	1314	660	29	23

**Table 4:** Digital User Experience Search

Digital Library	After Search	SC1	SC2	SC3	SC4	QA
IEEE	5293	2807	136	136	9	9
MDPI	1226	1101	1101	1101	3	2
Elsevier	9169	4730	867	81	6	6
Springer	12,003	5760	259	216	4	3
Wiley	3888	1704	104	102	2	2
Total	31,579	16,102	2467	1636	24	22

**Table 5:** 3D Conferencing Search

Digital Library	After Search	SC1	SC2	SC3	SC4	QA
IEEE	175	26	1	1	1	1
MDPI	11	9	9	9	1	0
Elsevier	3029	1375	280	82	4	3
Springer	3720	986	176	125	12	11
Wiley	35,067	6334	435	33	1	0
Total	42,002	8730	901	250	19	15

#### Data extraction

**Fig. 3:**
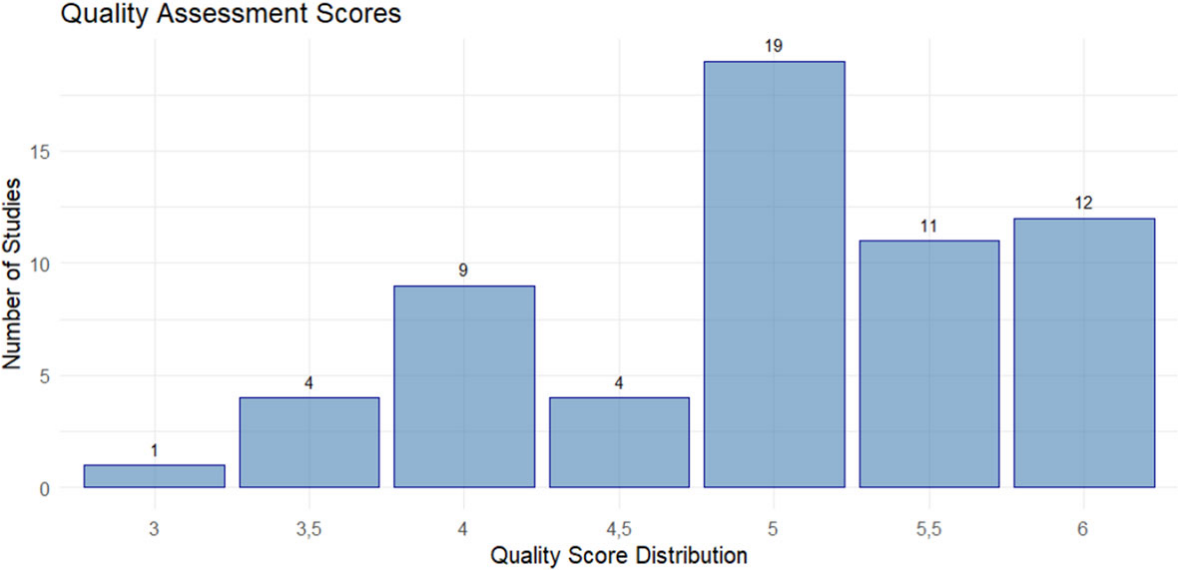
Quality Score Distribution

The data extraction process involves reading the 60 articles, graded by the quality distribution score, of which a distribution of the papers by score is displayed in Fig. 3. The selected papers are used to extract key features and obstacles relating to virtual conference and user experience design from the manuscripts. The data extracted relates to techniques employed, obstacles encountered and assists with answering the research questions. The collated data also caters for understanding and evaluating any emerging trends/patterns in the research. In order to undertake the data extraction process, a structured database is employed (in which articles are assigned a unique identifier) to ensure that the same data aspects are extracted from each of the research articles. Table 6 presents a sample of the dataset constructed following the extraction process (the actual dataset has high granularity). The data extraction process uncovered recurring trends (further outlined Section 3), 152 features and 146 obstacles.

**Table 6:** Data Extract Sample

Article ID	Keywords	Tools	UX Points	Targeted Domain	Obstacles	Features
VC1	Covid-19, disseminate, collaborations, feedback	WebEx, Web 2.0, Zoom	Lean	Medical	Multiple technologies	Scalable

#### Data synthesis

**Fig. 4:**
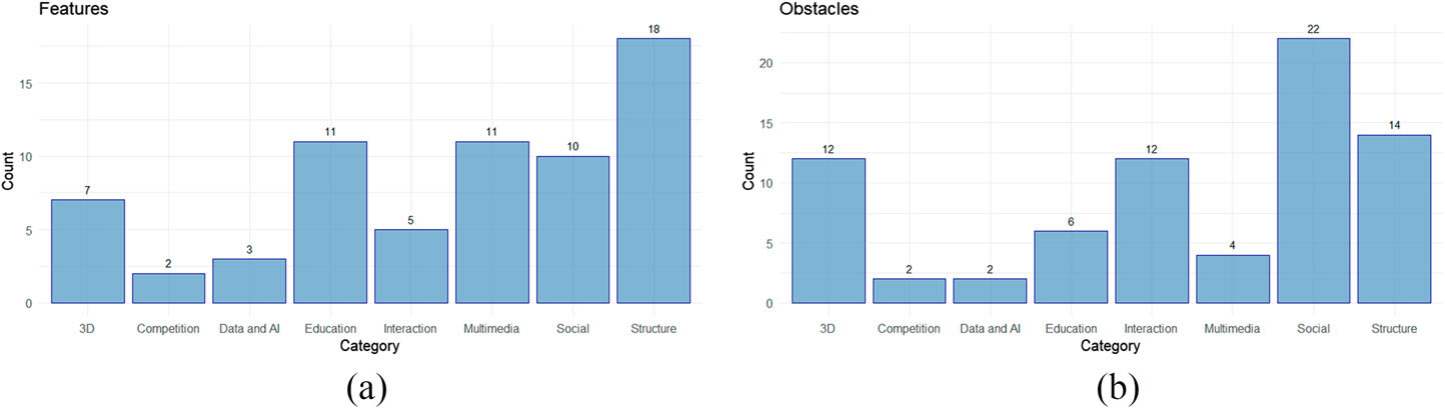
Category Distribution for **a** Features and **b** Obstacles

As in [66], the data synthesis process involves the collation of information ascertained during the data extraction process. As the manuscripts often have different terms for the same features, (e.g. computer-mediated communication [65] is within the virtual conference umbrella term), this process involves the use of collating synonyms and deciding on overarching concepts in order to group the features together despite the variations. In total, of the 152 features identified, the data synthesis process reduces the amount down to 67; with the 146 obstacles reduced to 74. Each of the features and obstacles are grouped into 8 categories, 3D, Social, Multimedia, Data and AI, Competition, Structure, Education and Interaction. A breakdown of the features and obstacles per category is displayed in Fig. 4.

## Results

This section first covers the main statistics concerning the 60 articles assessed in the SLR process. The section then moves on to answer the research questions identified in 2.2.1.

### General statistics

**Fig. 5:**
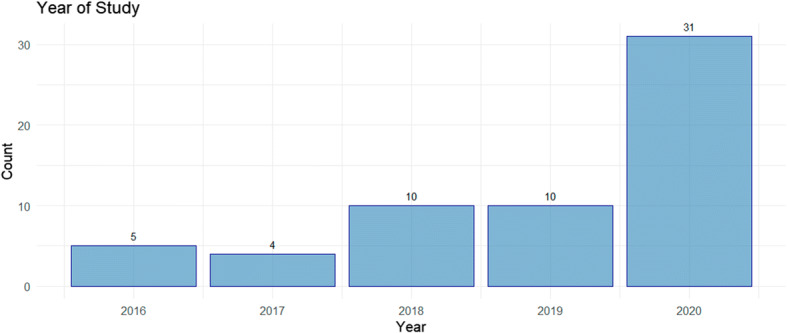
Year-wise Distribution of 60 Studies

The resulting 60 studies identified following the QA process, are presented in Table 7, with the year-wise distribution of the work displayed in Fig. 5. A higher number of open-access virtual-conference related works have been published in 2020 when compared with previous years.

**Table 7:** Primary Studies Following QA in Order of Search

[31]	[72]	[58]	[19]	[30]	[1]
[20]	[61]	[32]	[37]	[8]	[22]
[75]	[6]	[3]	[52]	[41]	[50]
[65]	[33]	[25]	[57]	[46]	[60]
[26]	[42]	[56]	[44]	[54]	[21]
[53]	[34]	[18]	[67]	[62]	[38]
[29]	[47]	[73]	[24]	[13]	[49]
[55]	[12]	[17]	[4]	[69]	[23]
[40]	[74]	[76]	[28]	[15]	[71]
[48]	[5]	[36]	[63]	[51]	[45]

**Fig. 6:**
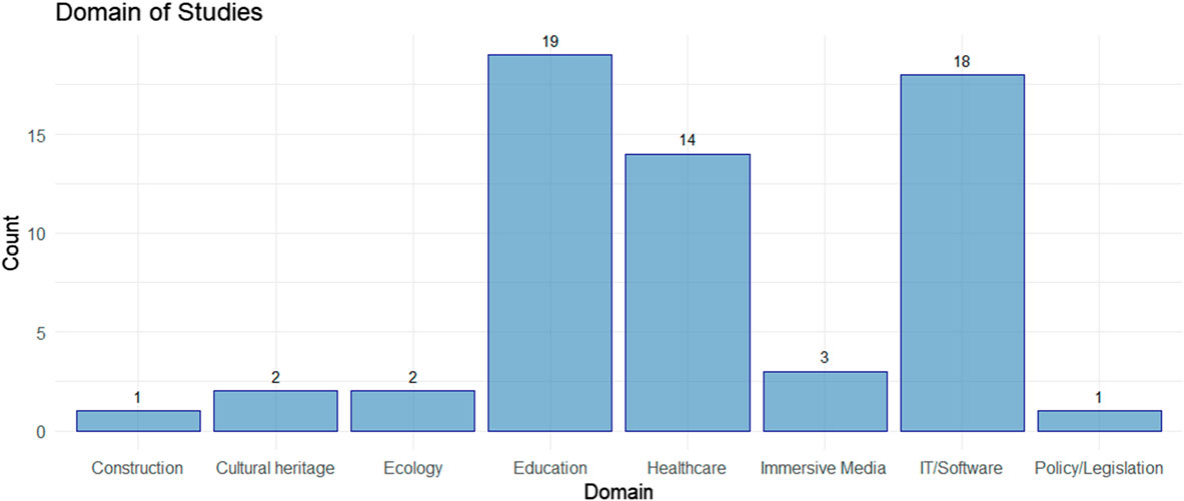
Domain of Studies

Figure 6 presents a visualisation of the publication domains, following the quality analysis process. The QA process produces 8 domains in which company-wide interaction, team discussions, screen sharing and face-to-face contact research takes place. In some cases, the theme of the article is for the betterment of virtual conferencing specifically; however, if this is the case the article is categorised within the IT/Software category. The Education, Healthcare and IT/Software research domains contained the highest number of publications.

**Fig. 7:**
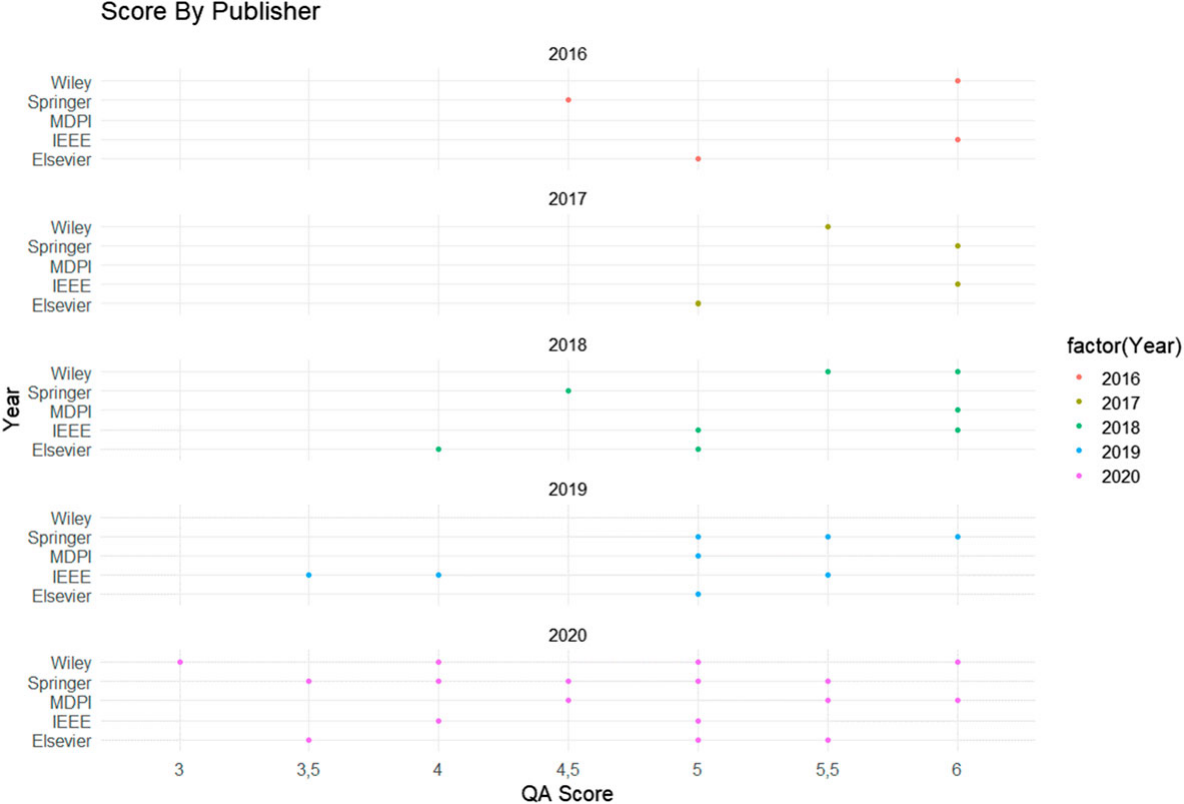
Score by Publisher

Further to Fig. 6, the score breakdown by journal publisher is presented in Fig. 7, which is also grouped by the publication year. The x-axis refers to the QA score for articles that scored 3 or above (the score distribution is outlined in Section 2, Fig. 3), and the y-axis details the digital repository where the article can be found.

As the graph displays, 2020 resulted in a higher number of virtual conference-related publications, but also more variety in the quality analysis. In the following section, the research questions outlined in Section 2.1.1 are addressed by means of a discussion into the underlying themes in the articles, in which features and obstacles were identified (as in line with the SLR standard, such in the work as by Tummers et al.).

#### Q1: What are the features and obstacles of current virtual conferencing platforms?

**Fig. 8:**
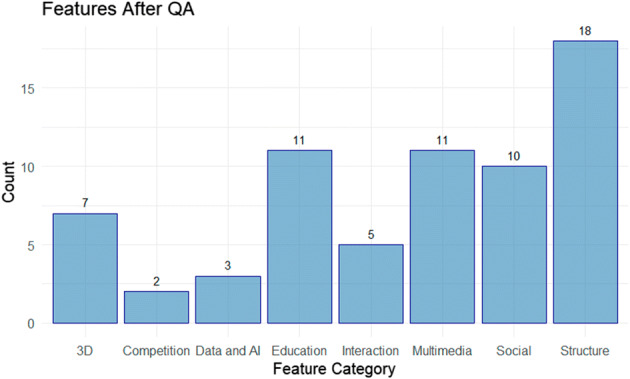
Feature Count after QA

The distribution of the 67 features is displayed in Fig. 8, with a full list presented alphabetically in Table 8 organised by the aforementioned categories (with 3D removed for discussion in 3.2.3). In some instances the features identified are ambiguous, and some features identified in a study are then found to be addressed as obstacles in others. For example, scalability identified as a feature in [34, 58], is referenced as an obstacle in other works [32]. This could be as a result of the differences between the technological requirements between 2D and 3D platforms. As some of the works use 3D technologies (including avatars, virtual reality and immersion) where scalability is an inherent challenge.

**Table 8:** Features Identified During QA

Social	Multimedia	Data and AI	Competition	Structure	Education	Interaction
Collaborative	Audio	Data Sharing	Leader boards (compare with others)	Autonomy	Educational	Avatars
Communicative	Mediated communication	Control Remote IoT	Rewards (e.g. points, achievement badges)	Dynamic/Flexible	Alternate Student and Instructor Views	Alternate Visual Reality
Goal-driven	Embedded	Al-Controlled Agents		Bespoke	Blended Learning	Haptic Feedback
Increased Outreach (International)	Content Sharing			Continuous Feedback	Hands-on Learning / Training	Immersive
Social Network-Integrated	Moving			Free	Individualized	Hybridised
Accessible	Recorded			Increased Efficiency	Immediate Feedback	
Maintain Social Relationships	Remote Manipulation			Lowered Carbon Emissions	Intercommunication with Lesson	
Meeting format can address inequalities in participation	Shared Viewpoint (Virtual Camera)			Many-to-many	Learning Support	
Multiple Applications in One	Text			Many-to-one	Full Control for Teachers	
Synchronous	Video engagement			Monetized	Unique Learning	
	Virtual Multi-Board			One-to-many	Highly Interactive	
				One-to-one		
				Scalable		
				Sophisticated Security		
				Stared Projects		
				Cloud-based		
				Real-time		
				Administrative		

Flexibility is listed as a fundamental feature for the structure of the virtual conference. However, the exact specification of what flexibility refers to is ambiguous. Sweetman et al. for example, discuss that accommodating diverse and evolving student scenarios is beneficial [60], and this could be classed as a flexibility feature. Other works document flexibility as a necessity without detailed elaboration [65, 69]. However, features such as bespoke, hybridised, blended learning, collaborative and autonomous may provide an insight into the need for flexibility. Furthermore, as Schouten et al. discuss, there is a need to allow individual users to indicate their own learning styles [54], meaning flexibility is required in the conference platform to customise the learning or communication environments for both the teacher and students [48].

Core features recur throughout many of the articles including, audio, text, content sharing, engagement, free and social interaction. However, some works refer to other more unique features, such as competition, autonomy and reward systems. As Sardi et al. discuss, by introducing competition and a reward system (e.g. badges and points) and leader boards, effective solutions for learning-based environments [53] the need for continuous feedback in other domains could also be made possible.

Regarding extraction of the obstacles found in the articles, examples include synchronous delivery [4], cybersickness [3], immersion quality [25], scalability [32] and avatar personalisation [26]. All obstacles identified in the SLR for all virtual meeting contexts, are presented alphabetically in Table 9, again organised into the aforementioned categories.

**Table 9:** Obstacles Identified for all Virtual Meeting Contexts During QA

Social	Multimedia	Data and AI	Competition	Structure	Education	Interaction
Accessibility (Technology)	Audible Modules	Accurate Information	Reward System	Third Party-Tech has Limited Control/Alteration for Owner	Student Behaviors are Anticipated Rather than Observed	Avatar Personalisation
Acceptability	Acknowledgments	Vulnerability of the Final Code	Track Progress	Technology Integration	Blended Learning	Immediate / Real-Time
Communication in User-directed Simulations	Synchronous Delivery			Cost for Better Tech (e.g. zoom)	Broad Range of Course Structures / Diversity of Content	Interaction Techniques
Basic Need Satisfaction	Resolution			Country-Based Regulations for Digital Content	Direct Supervision	Both Enjoyable and Engaging
Isolated using HMD				Customisation	Disability Support	Immersion Quality in VR
Co-collaboration activities				Degree of Polish	Lack of hands-on learning	Scalability
Conveying Mutual Gesture/Emotion Cues				innovation		Subjective Immersion
Culture				Access to Tech/Tools		VR equipment access
Daily Reviews				Maintenance Costs		Poor Experience with Quick Movements in VR
Decreased Motivation				Multiple Technologies		Cybersickness
Distributed Pattern of Conversation				Notifications/Alerts		Multiuser Environment Interaction
Inclusion				Perceptual Quality		Digital Interface
ICT Skills and Access				Performance		
Lack of Non-Verbal Communication (Eye Contact)				Usability		
Limited UX						
Loss of Most Active User						
Moderator Requirement						
Multiple Networks of Practice						
User Responsibilities						
Screening Participants						
Social Influence (variety)						
Subjective experience						

#### Q2: What are the user experience considerations?

41 user experience considerations are identified in the literature survey, as outlined in Table 10, again, presented alphabetically. Two notable recurring considerations are the need for better presence (a feeling of being there in a perceptible external world around the self) [37] during a virtual conference session and the lack of being able to see physical gestures (and eye contacts) [8]. For example, as Cai et al. discuss, social cues affect remote communication, and an additional camera may be appropriate in order to provide a viewing perspective, which would allow for the conveyance of mutual gesture cues [8].

**Table 10:** User Experience Considerations

User Experience Considerations			
Adaptable	Ease of interaction	Incompleteness	Reduced Waiting Time on Start-up
Ambient Intelligence	Efficient	Interactive	Regions of Interest Areas
Awareness tool/interface	Empathy	Intimacy	Reliable
Cloud Architecture	Environmentally Conscious Design	Lean	Responding to User Needs
Code of conduct	Extended Reality	Mobile	Structured
Computer-Guided	Gesture Detection	Multimodal	Tactile
Decentralised	High Computer Memory (1–3 GB)	Presence	Untethered
Design Style that Reuses Web Technologies	High-Speed Internet Connection	Puppeteered (using users’ keyboard, mouse, or controller inputs)	User-Friendly Interface
Digitally Inclusive	Human Computer Interaction	Quality of Experience	Visual Attention
Duration	Immediacy	Realism of the image	Well-balanced (synergy between representation and experience)

**Fig. 9:**
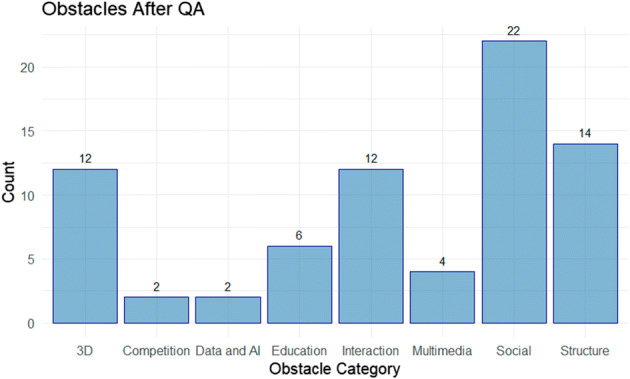
Count for Obstacles Following QA

In addition to the above considerations, 74 obstacles are identified. The distribution of the obstacles across the 8 categories outlined in Section 2.2.6 is displayed in Fig. 9. The highest volume of obstacles is related to social considerations. This would be in line with the user experience design issues identified above, which outline the need for better social cue integration into virtual conference platforms.

Table 10 details the full list of obstacles. Digital inclusion is a recurring challenge within the works identified. Access to technology and the skills required to operate tools (e.g. haptic control devices [3]) and equipment (e.g. high-speed internet [5] and VR-gear) are natural boundaries for the end-user when working with virtual conferencing solutions. However, technology must also cater for users with disabilities such as deuteranopia, autism, intellectual disability, emotional disturbance, etc. [24]. This is more so the case when the technology involves communication in a virtual conference when VR is employed or interacting with screen-based 3D conference environments.

#### Q3: What are the digital considerations for 3D virtual conference applications?

Regarding 3D conferencing, completed 3D models are identified as a critical issue. Incomplete 3D models can produce a disturbing experience for users [18]. Also within a 3D-based environment, puppeteering may be required to allow the user to control their virtual avatar [32] when negotiating the virtual environment. For example, using the keyboard and mouse separately to move different body parts would allow the user to integrate physical gestures when communicating.

However, access to technology for the conference provider is also a challenge. When developing a 3D conference solution a cloud-based infrastructure is inevitable to provide scalability and address the need for real-time interaction. For that reason, 5 of the 60 works reference existing tools such as Second Life for the virtual environment applications [5, 13, 26, 37, 62]. In this case, the backend infrastructure and 3D world are already created, meaning the developer is benefitting from the use of third party technology. However, this also means that control over the environment is limited and customisation may be restricted. This issue of lack of control is identified as a potential consideration by Ding et al. [17]. Table 11 presents the full list of 3D considerations divided into features and obstacles.

**Table 11:** Considerations for 3D in virtual conference applications

Features	Obstacles
360 Degree videos	3D Content Production
Accurate representation of indoor environment	3D Animation Production
Harness BIM	BIM Files Have Large Geometry
Digital gaming	Field of View Awareness
Digital twin	Continuous Improvement Requirement
Virtual humans	Need for dedicated device(e.g. Game Controller)
Virtual Reality	Fluidity
	Quality and Realism
	Scanning Requirements
	Texture Parameters
	Camera FOV
	Decreased Frame Rate

VR is a common approach for next-generation 3D virtual conference solutions, and 20 of the 60 articles reference the technology as a potential education, training and communication solution moving forwards. However, Liu et al. discuss that negative immersive experiences can emerge when a user moves too quickly in VR [35] and other challenges, such as cybersickness, need to be addressed before wider deployment [3]. Yet, 3D and VR are remain prominent technologies for immersive training, and cultural heritage communication applications [12] particularly.

### Discussion

Section 3.1 provides a statistical overview based on an SLR analysis, concentrating the search on IEEE Xplore, MDPI, Elsevier, Springer, Wiley digital libraries. To the best of our knowledge, this article presents the first SLR analysis of virtual conference solutions that integrates 3D into the investigation. With a total of 391,580 articles identified, 60 were then selected for data extraction and discussion based on a comprehensive selection process and quality assessment. The subsequent analysis of the 60 articles provides 67 features and 72 obstacles, which can be of value for virtual conference technology developers in creating their software applications. Both the features and obstacles were divisible into 8 categories: 3D, Competition, Data and AI, Education, Interaction, Multimedia, Social and Structure.

However, one further contribution to knowledge is discussion of the recurring underlying themes within the 60 articles. One of the most prominent is Covid-19, which is often argued as a motivator or catalyst for the research taking place. As discussed in the introduction, the trend towards working from home and virtual conference solutions to facilitate this was already under-way pre-pandemic, but the pandemic has acted as a catalyst speeding up this process. Yet, covid-19 has created a somewhat prominent line of focus within the articles in the present period (and its peculiar requirements and problems). To name a few examples, Lamming et al. discuss the use of an online seminar series as a result of the Covid-19 pandemic affecting regional and national meetings [31]; Milovanović et al. outline that Covid-19 has brought considerable challenges resulting in the need for the emergency design of education material [42]; and Rubinger et al. detail that the Covid-19 pandemic has created a need to maximise communication within the medical and scientific community [52]. Out of the 60 articles, 15 reference Covid-19. This could be the core reason for why a higher number of articles are present from 2020 in the search results compared with the previous four years. Again, the domain of the articles is relating majorly to healthcare, education and IT, which would be logical due to the impact Covid-19 has had on education, training and the medical infrastructure.

Other recurring trends include virtual interviews, virtual training, digital reliance, gamification and social connections. Culture is also a recurring keyword within the articles for two reasons, some of the articles are related directly to digital cultural heritage applications [12]. However, in other cases the works refer to the need to virtual conference tools to embrace a solution for the lack of workplace culture when working from home, particularly for medicine education [20]. It is, of course, a challenge to recreate a workplace culture within a digital setting, or recreate a learning culture that a student would find typically in a physical classroom environment. Yet, the investigation uncovered features that are beneficial to the virtual digital conference experience. Whilst this (the need for the creation of culture) was an unexpected finding, it would a logical consideration as the need for meeting in person, for example at a physical international conference, has benefits beyond the access the knowledge sharing. Considerations, such as this, may be easy to overlook when developing a virtual conference application, but they are crucial insights for both global virtual teams literature and virtual teams or those in educational and management positions, which emphasises the importance of regular SLR investigations that survey the existing landscape.

The authors also emphasise that the aims and findings depicted in the paper are not intended to promote the use virtual conferencing solutions over other approaches or dissuade from their use, but rather showcase the existing features and obstacles associated with existing software as is found in current open access literature only. This is, of course, also a limitation of the work, as it may be the case that some prominent paid-for articles have unique features or obstacles that would benefit this investigation. Yet, the adoption of the open-access approach in this article is intentional in order to make this research repeatable and, similarly, the findings discussed from the related articles available to as wide an audience as possible. This means others will be able to review the examined references. It is also evident that the volume of open-access research articles related to virtual conference applications has increased sufficiently over the last five years to provide a detailed and structured SLR output.

## Conclusion

The Covid-19 pandemic has had a disruptive impact in 2020, but it has also created opportunities for virtual conference creators to develop solutions to support remote working, but the pandemic has acted as a catalyst to bring the notion of virtual conferencing more into the mainstream. Virtual conferencing may also remain part of our work environment for the foreseeable future; with authors such as [23] and [21] arguing that virtual conferencing is becoming a preferred solution for those wishing to reduce their carbon footprint, meaning virtual conference development opportunities may extend beyond pandemics as conference participation is an essential aspect for the development of research and creation of novel ideas. Therefore, immersive virtual conferencing may be an ideal solution, if yet a challenge.

In this article, an investigation into the features and obstacles found within virtual conference solutions (both 2D and 3D) are discussed. Academic literature repositories were the primary target of the investigation, however, this could also be expanded to include other sources, such as blogs and other grey literature in future investigations, where there are often software applications not considered in academic publications. In addition, paid-for articles were not part of the SLR investigation, which is a limitation and it is possible that articles suitable for discussion were omitted from the findings. However, an open-access review approach was adopted to cater for repeatability of the research and for direct access to the findings from the articles discussed in the SLR. Our approach also means it is possible to expand the investigation by the inclusion of such articles in future studies. In our future work, we will devise a decision model framework to help others plan and setup a feature-driven conference solution based on the data collated in the SLR presented in this article. Future directions of the research could also include survey-based studies of existing solutions to a wide group of end-users or duplications of the investigation using paid for articles only to provide a comparison of the findings.

## Footnotes

1 https://www.tudelft.nl/en/education/information-and-experience/tu-delft-campus-in-minecraft/

2 https://www.educationandlearning.nl/news/minecraft-campus-update-upcoming-features
